# 

**Published:** 1978

**Authors:** 


NATIONAL LIBRARY

S 30 NOV 1979 Z

OF MEDICINE

### BRISTOL MEDICO-CHIRURGICAL JOURNAL

JANUARY/APRIL 1978
VOLUME 93 (i/ii)
NUMBER 345/346

